# Structural Variations of Vaginal and Endometrial Microbiota: Hints on Female Infertility

**DOI:** 10.3389/fcimb.2020.00350

**Published:** 2020-07-14

**Authors:** Lucia Riganelli, Valerio Iebba, Mariagrazia Piccioni, Isabella Illuminati, Giulia Bonfiglio, Bruna Neroni, Ludovica Calvo, Antonella Gagliardi, Massimo Levrero, Lucia Merlino, Marianna Mariani, Oriana Capri, Daniela Pietrangeli, Serena Schippa, Francesca Guerrieri

**Affiliations:** ^1^Department of Experimental Medicine, Sapienza University of Rome, Rome, Italy; ^2^SSD of Advanced Microbiology Diagnosis and Translational Research, Institute for Maternal and Child Health-IRCCS “Burlo Garofolo”, Trieste, Italy; ^3^Department of Medical, Surgical and Health Sciences, Azienda Sanitaria Universitaria Giuliano Isontina (ASU GI), Trieste, Italy; ^4^Department of Obstetrics and Gynaecology, Sapienza University of Rome, Rome, Italy; ^5^Department of Internal Medicine and Medical Specialties, Sapienza University of Rome, Rome, Italy; ^6^Department of Public Health and Infectious Diseases, Sapienza University of Rome, Rome, Italy; ^7^Center for Life NanoScience@Sapienza, Istituto Italiano di Tecnologia, Rome, Italy; ^8^Epigenetics and Epigenomic of Hepatocellular Carcinoma, Cancer Research Center of Lyon (CRCL), UMR Inserm 1052 - CNRS 5286, Lyon, France

**Keywords:** infertility, ART, microbiota, endometrium, vagina, bacteria, NGS

## Abstract

Microbiota are microorganismal communities colonizing human tissues exposed to the external environment, including the urogenital tract. The bacterial composition of the vaginal microbiota has been established and is partially related to obstetric outcome, while the uterine microbiota, considered to be a sterile environment for years, is now the focus of more extensive studies and debates. The characterization of the microbiota contained in the reproductive tract (RT) of asymptomatic and infertile women, could define a specific RT microbiota associated with implantation failure. In this pilot study, 34 women undergoing personalized hormonal stimulation were recruited and the biological samples of each patient, vaginal fluid, and endometrial biopsy, were collected immediately prior to oocyte-pick up, and sequenced. Women were subsequently divided into groups according to fertilization outcome. Analysis of the 16s rRNA V4-V5 region revealed a significant difference between vaginal and endometrial microbiota. The vaginal microbiota of pregnant women corroborated previous data, exhibiting a lactobacilli-dominant habitat compared to non-pregnant cases, while the endometrial bacterial colonization was characterized by a polymicrobial ecosystem in which lactobacilli were exclusively detected in the group that displayed unsuccessful *in vitro* fertilization. Overall, these preliminary results revisit our knowledge of the genitourinary microbiota, and highlight a putative relationship between vaginal/endometrial microbiota and reproductive success.

## Introduction

The human body is colonized by billions of microbes including bacteria, archaea, fungi, viruses, and phages (representing 90% of the human viroma) (Gagliardi et al., 2018), with a coding capacity 150-fold higher than that of eukaryotic cells (Sender et al., 2016). Furthermore, 80% of the human microbiota resides in the intestinal tract, which is considered to be an additional human organ (O'Hara and Shanahan, 2006; Schippa and Conte, 2014). Indeed, the gut microbiota strongly impacts our health by hindering pathogen colonization (O'Hara and Shanahan, 2006), exerting metabolic and trophic functions, and contributing to the development of the immune system (Belkaid and Harrison, 2017). A further 9% of the human microbiota is found in the urogenital tract (Sirota et al., 2014). Lactobacilli represent 90–95% of vaginal bacteria, the four major species being *Lactobacillus crispatus (L. crispatus), Lactobacillus iners (L. iners), Lactobacillus jensenii (L. jensenii)*, and *Lactobacillus gasseri (L. gasseri)* (Antonio et al., 1999; Pavlova et al., 2002; Vásquez et al., 2002; Zhou et al., 2004; Shi et al., 2009). Their ability to produce lactic acid, hydrogen peroxide (H_2_O_2_), bacteriocins and probiotics, contributes to a healthy genitourinary status, as these are hostile conditions for many microbes, including pathogens (Skarin and Sylwan, 1986; O'Hanlon et al., 2013). The most common causes of variations in the vaginal microbiota composition are the menstrual cycle, sexual activity, and changes in the intestinal ecosystem (e.g., constipation or diarrhea). Alterations of vaginal microbiota could favor the onset of several pathologies such as bacterial vaginosis (BV), characterized by a reduction of lactobacilli and abnormal growth of anaerobic bacteria and associated with an increased risk of acquiring human immunodeficiency virus (HIV) (Verstraelen and Swidsinski, 2013). It has been suggested that the vaginal microbial ecosystem is more stable when dominated by a prevalence of *L. crispatus* compared to those dominated by *L. iners* or a mixture of lactobacilli (France et al., 2016). Recent studies identify several bacterial species as biomarkers of cervical-vaginal microflora to improve both accuracy of infertility diagnosis and therapeutic procedures (Cook et al., 2009; Campisciano et al., 2017). *L. iners*, in particular, is described as an important biomarker of vaginal microbiota health able to predict the outcome of Assisted Reproductive Technology (ART) (Tärnberg et al., 2002; Vásquez et al., 2002). Chen et al. showed different microbial communities among vagina, cervical canal, uterus, fallopian tube and peritoneal fluid, demonstrating a non-sterile environment in the female reproductive tract (Chen et al., 2017), disproving the initial dogma that a healthy uterine cavity was sterile, and that the presence of microbes was a sign of pathology (ascension of bacteria through the cervix, through blood, or for gynecologic procedure like ART or insertion/removal of intrauterine devices). It is now well-established that the uterus hosts a specific flora and recent evidence has emerged indicating that the uterine microbiota impacts female reproduction, health, and disease. However, the composition of the uterine cavity microbiota is still controversial and its role remains highly debated (Baker et al., 2018; Simon, 2018; Peric et al., 2019). Several groups define two kinds of uterine microbiota: the *Lactobacillus*-dominated (>90% lactobacilli), and the non-*Lactobacillus*-dominated (<90% lactobacilli with >10% of other bacteria), the latter being related to a significant decrease in *in vitro* fertilization (IVF) and live birth rates (Moore et al., 2000; Moreno et al., 2016). Another study analyzing endometrial samples of 19 women, reported no lactobacilli colonization but a predominant bacteroidetes and proteobacteria environment (Verstraelen et al., 2016). An additional paper showed no correlation between lactobacilli concentration and pregnancy in IVF patients, exposing *Flavobacterium* spp as the most abundant species in the uterine microbiota (Franasiak et al., 2016). The different results obtained on uterine microbiota could be due to the high rate of contamination from adjacent vaginal tissue depending on sampling modalities (Baker et al., 2018). Vaginal and uterine microbiota, as well as the immune and cytokine environment, influence the outcome of conception in both natural reproduction and in IVF (Robertson et al., 2011; Campisciano et al., 2017). It was suggested that even a small inflammatory response in the endometrium could compromise embryo fitness or prevent its implantation (Moore et al., 2000). A better understanding of the influence of the uterine microbiota on embryo implantation, and its relationship with the adjacent vaginal bacteria, could help us identify changes in RT microbiota composition having a negative impact on reproductive outcome. The aim of our study was to explore structural variations of vaginal and endometrial microbiota, in an attempt to define possible biomarkers related to embryo implantation failure. To this purpose, we characterized vaginal (cytobrush) and endometrial (biopsy) microbiota from asymptomatic and infertile women undergoing ART, immediately before ovule collection and after hormonal stimulation.

## Materials and Methods

### Study Population

Thirty-four Caucasian women, aged 22–43 (median age 37) were consecutively enrolled at the Infertility Department of the University of Rome La Sapienza, Italy, between April 2017 and April 2018. This group was further subdivided into four groups according to their age, namely ranging from 22 to 31 (6/34), 32 to 37(14/34), 38 to 40(4/34), and 41 to 43 (10/34) years. Infertile patients of reproductive age were recruited, including nulliparous women and women with previous pregnancies, regardless of their outcome. Each patient had previously undergone an assisted reproductive technology (ART) of second level (*in vitro* fertilization and embryo transfer or intracytoplasmic sperm injection) with implant failure. We included patients with infertility related to tubal occlusion (7/34), endometriosis (3/34), ovulatory disorder (9/34) or idiopathic infertility (13/34) for at least 1 year. For each woman, demographic, and clinical-anamnestic data were collected, with particular attention to gynecological, obstetric, and pathological history (Table 1). Among the four pregnant patients (group A), two suffered from Hashimoto's thyroiditis and were treated with levothyroxine in good endocrine condition (thyroid stimulating hormone level under 2.5 mu/L) before conception, a third patient reported levothyroxine replacement therapy following total thyroidectomy. Moreover, two of these patients had laparoscopy due to stage IV endometriosis, one had operative hysteroscopy for U3 uterine anomaly, and one had hysteroscopy for G1 fibroid. Surgery was performed at least 12 months before the ART cycle was initiated. The exclusion criteria were: male factor (an impact on implantation rate independent of the female patient), risk of pelvic inflammatory disease (positive swabs in the last 3 months of enrolment for *Neisseria gonorrhoeae, Chlamydia trachomatis, Mycoplasma hominis, Mycoplasma genitalium*, or *Ureaplasma urealyticum*), cervical carcinoma, ongoing pregnancy, anomalous uterine bleeding, and subject of intrauterine procedures in the 6 months preceding the study that could affect the integrity of the uterine microbiota. In addition, data about the ART cycle to which patients will be subjected according to the methodology used routinely in our center were collected. All women were submitted to a mild/minimal stimulation protocol of recombinant FSH (rFSH) combined with GnRH antagonist. All woman recruited displayed no side-effects to the stimulation protocol (such as hyperstimulation syndrome or absent response to the hormonal therapy) and all of them reached the embryo-transfer phase. The study was conducted according to the ethical standards expressed in the Declaration of Helsinki. It was approved by the ethics committee of the University of Rome “Sapienza” (protocol number: AR11715C81FB56F4) and all subjects provided written informed consent.

**Table 1:** Patient demographics.

**Total cases (34 pts)**	**Pregnant group (A) (4 pts)**	**No- Pregnant group (B) (30 pts)**
Age range (mean)	31–42 (36)	22–43 (34,6)
22–31	1	5
32–37	1	13
38–40	1	3
41–43	1	9
Cause of infertility (*n*)		
-idiopathic	2/4	11/30
-endometriosis	-	3/30
-tubal factor	1/4	6/30
-ovulatory disorder	1/4	8/30
Medical treatment (Eutirox, Tirosint, Antihistamines) (%)	3 (75%)	6 (20%)
Previous gynecological surgery (%)	4 (100%)	22 (73.3%)
Smoking habits (<10 daily) (%)	3 (75%)	12 (40%)
Parity (%)	/	8 (26.6%)
Previous miscarriage (%)	/	5 (16.6%)

### Sample Collection

The biological samples of each patient were taken at day 21 of the menstrual cycle before starting the personalized stimulation protocol. During the echo-guided pelvic procedure, the patient assumed the lithotomy position; the external genitals were disinfected, the sterile vaginal speculum was inserted and the vaginal fluid was collected by Cytobrush by rotating 360° in the posterior fornix. Subsequently, the cervix was cleaned with physiological solution (NaCl 0.9%) and the intrauterine insemination catheter (Artacath™ ©) was introduced through the cervical canal into the uterine cavity. The Artacath catheter has an echo-marker which allows the insertion of the catheter 1 cm from the internal uterus orifice. During the procedure, the Pipelle catheter was covered by the Artacath catheter, meaning that the Pipelle was completely protected from contamination during the passage through the vagina and cervical canal. The introduction through the cervical canal of the Artacath catheter (diameter 4 mm), is considered to guide the gentle introduction of the Pipelle catheter (diameter 2.5 mm). The Artacath catheter is commonly used for intrauterine insemination and its diameter is perfect to allow the insertion of the Pipelle catheter. Based on the combined use of these two catheters the procedure could therefore be considered almost sterile.

Indeed, the endometrial samples were collected by the Pipelle catheter, using an ultrasound guide, 2 cm away from the Artacath catheter. Therefore, any possible contamination that may arise from the end of the catheter is avoided.

This method is necessary in order to avoid the possible contamination from the colonized lower tract, because even minor contaminants could render our analyses of the microbiota uninterpretable. In the uterine cavity, under negative pressure, the Pipelle was used back and forth four times, rotating 90° each time and then extracted. Endometrial biopsies were collected in labeled sterile tubes (Falcon 50 mL) containing protected tissue reagent (Allprotect Tissue reagent, Qiagen), for immediate stabilization of DNA, RNA and proteins in human tissues, and stored at −80°C until further use. The patients enrolled in the study were further divided into two groups on the basis of the success their ART cycle, the first group characterized by women who achieved pregnancy, and the second by women who remained infertile.

### Total DNA Extraction and NGS Sequencing

Total DNA was extracted from all collected samples using dedicated kits (DNeasy Blood and Tissue kit, cat#69506, Qiagen, Hilden, Germany) following the manufacturer's instructions. To achieve maximum yield from Gram-positive bacteria, an additional step was added in the DNA purification protocol. Briefly, a 2-h incubation step with 2 mg/mL of lysozyme (cat# L6876, Sigma-Aldrich, Milan, Italy) at 37°C was performed, and incubation times were doubled in order to increase DNA yield. The extracted DNA was quantified using the Nanodrop Spectrophotometer 2000c. DNA integrity was visualized by 1% agarose gel electrophoresis containing 0.5 mg/mL ethidium bromide (EtBr). A defined DNA concentration (5–10 ng/μL) for all samples was used for the library preparation. Samples were subjected to robotic (Maxwell® RSC Instrument, Promega, Wisconsin, USA) PCR amplification, library preparation, and sequencing according to the Illumina 16S metagenomics standardized operational workflow for the 16S rRNA V3-V4 region (16S Metagenomic Sequencing Library Preparation, Part # 15044223 Rev. B). Appropriate blanks (negative controls) and a mock microbial DNA community standard (ZymoBIOMICS Microbial Community DNA Standard D6305) was used to control library preparation and sequencing (Supplementary Figure 1). Each 16S library was checked for size with an Agilent 2200 Tapestation (Agilent Technologies, Santa Clara, CA, United States) and quantified with a Qubit 2.0 fluorometer using the Qubit dsDNA HS Assay Kit (cat# Q32851, Thermo Fisher Scientific, MA, United States). Sequencing was performed at the Italian Institute of Technology (https://www.iit.it/it/centers/clns-sapienza) with an Illumina MiSeq platform, Reagent Kit v3 (cat# MS-102-3003, Illumina, San Diego, CA, United States), 2 × 300 paired ends.

### OTU Species Assignment

Raw FASTQ files were analyzed with Mothur pipeline v.1.39.5 for quality check and filtering (sequencing errors, chimera) on a Workstation DELL T7910 (Round Rock, Texas, United States). Raw reads (5,996,367 in total, on average 88,182 per sample) (Supplementary Text 1) were filtered (1,008,661 in total, on average 17,696 per sample) and clustered into Operational Taxonomic Units (OTUs), followed by the elimination of low-populated OTUs (till 5 reads) and by *de novo* OTU picking at 97% pair-wise identity using standardized parameters and SILVA rDNA Database v.1.19 for alignment. Overall, considering vaginal and endometrial samples, 319 bacterial species were identified. Sample coverage was computed with Mothur and was on average higher than 99% for all samples, thus confirming the suitability of the normalization procedure for subsequent analyses. Bioinformatics and statistical analyses on recognized OTUs were performed with Python v.2.7.11. The most representative and abundant read within each OTU (as evidenced in the previous step with Mothur v.1.39.5) underwent a nucleotide Blast using the National Center for Biotechnology Information (NCBI) Blast software (ncbi-blast-2.3.0) and the latest NCBI 16S Microbial Database (ftp://ftp.ncbi.nlm.nih.gov/blast/db/). A matrix of bacterial relative abundances was built at each taxon level (phylum, class, order, family, genus, species) for subsequent multivariate statistical analyses.

### Statistical Analysis

Only species having a prevalence (independently of their relative abundance) higher than or equal to 20% were considered (*n* = 18), except for drawing pie charts where all species (*n* = 319) were considered, though in the graphical representation all species having a relative abundance <0.5% were collectively reported within the category “Other.” Raw matrix (tabular) data were first normalized then standardized using QuantileTransformer and StandardScaler methods from Sci-Kit learn package v0.20.3. Normalization using the output_distribution=“normal” option transforms each variable to a strictly Gaussian-shaped distribution, whilst the standardization results in each normalized variable having a mean of zero and variance of one. These two steps of normalization followed by standardization ensured the proper comparison of variables with different dynamic ranges, such as bacterial relative abundances. Measurements of α diversity (within sample diversity) such as observed_otus and Shannon index, were calculated at OTU level using the SciKit-Bio package v.0.4.1 (Schloss and Handelsman, 2007). Exploratory analysis of β-diversity (between sample diversity) was calculated using the Bray-Curtis measure of dissimilarity calculated with Mothur and represented in Principal Coordinate Analyses (PCoA), while for Hierarchical Clustering Analysis (HCA) “Bray-Curtis” metrics and “complete linkage” method were implemented using custom scripts (Python v.2.7.11) (Schloss and Handelsman, 2007; Buttigieg and Ramette, 2014). ANalysis Of SIMilarity (ANOSIM) or PERMutational ANalysis Of VAriance (PERMANOVA), both measuring the difference in dataset centroids, were calculated after 999 permutations with SciKit-Bio package v.0.4.1. We implemented Partial Least Square Discriminant Analysis (PLS-DA) and the subsequent Variable Importance Plot (VIP), with a leave-one-out method (LOO) permuted a number of times equal to the number of samples within the overall cohort, as a supervised analysis in order to identify the most discriminant bacterial species among the different cohorts. Reported VIP values for each bacterial species are the mean of the permuted VIP values after the LOO method. Mann-Whitney *U* and Kruskal-Wallis tests were employed to assess significance for pair-wise or multiple comparisons, respectively, taking into account a *P* ≤ 0.05 as significant. Fisher's test with Freeman-Halton extension (Freeman and Halton, 1951) was performed where requested. LEfSe analysis (Segata et al., 2011) was employed on bacterial species relative abundances which were statistically different after Mann-Whitney *U*-test (for pairwise comparison). Benjamini-Hochberg two-stages false detection rate (FDR) at 10% was then applied, with the additional constraint of leaving only the species which were represented by at least five data points.

### Network Analysis

Cross-correlation Pearson matrices for network analysis (metric = Bray-Curtis, method = complete linkage) were generated with in-house scripts (Python v.2.7) and visualized with Gephi v.0.9.2, considering species having a prevalence ≥20% and a significant Pearson correlation coefficient divided into eight categories to define edge thickness (Li et al., 2008). A network analysis was performed on each dataset using co-occurrences and concomitant significance of pair-wise Pearson correlation coefficient, taking care of an optimized visual representation as proposed by current guidelines (Merico et al., 2009; Faust and Raes, 2012; Faust et al., 2012; Lozupone et al., 2012; Berry and Widder, 2014). The degree value, measuring the in/out number of edges linked to a node, and the betweenness centrality (a measure of the “keystoneness”), measuring how often a node appears on the shortest paths between pairs of nodes in a network, were computed with Gephi v.0.9.2. Intranetwork communities were retrieved using the Blondel community detection algorithm by means of randomized composition and edge weights, with a resolution equal to 1 (Blondel et al., 2008; Lambiotte et al., 2014).

### Data Availability

Raw data (fastq.gz files) are available at the National Center for Biotechnology Information (NCBI) Sequence Read Archive (SRA) database under the Bioproject PRJNA603234.

## Results

### Differences in Composition Among Endometrial and Vaginal Microbiota

**Figure 1:**
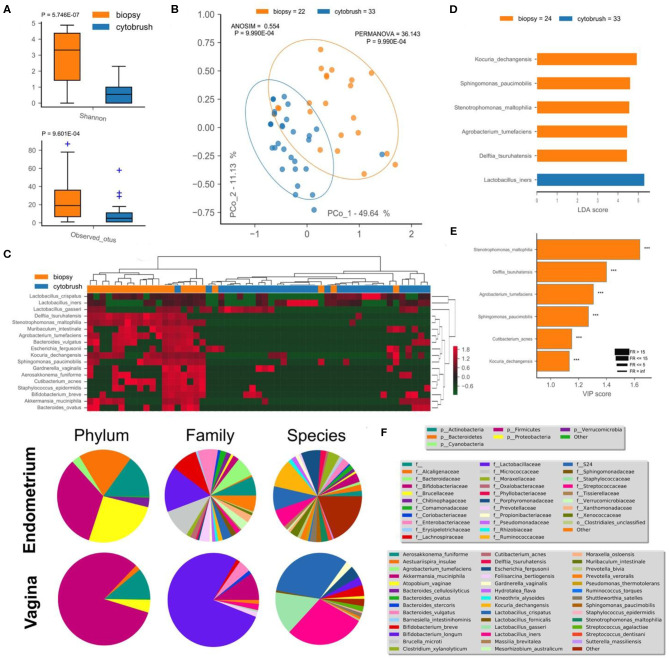
Endometrial (biopsy) and vaginal (cytobrush) tissues harbor a different microbiota composition. Alfa-diversity measurements of biodiversity (Shannon) and richness (observed OTUs) were assessed **(A)**, along with beta-diversity (unsupervised PCoA—**B**, unsupervised HCA—**C**). Percentage of variance embraced by each new coordinate is reported in percentage for each axis. Ellipses describing the 95% of confidence are depicted for each cohort. ANOSIM and PERMANOVA metrics were implemented with 999 permutations to assess differences. Hierarchical Clustering Algorithm (HCA) and Principal Coordinate Analysis (PCoA) were based on Bray-Curtis distance metrics and normalized/standardized bacterial relative abundances. Supervised LEfSe **(D)** and PLS-DA VIP **(E)** were used to find bacterial biomarkers. Variable Importance Plot (VIP) was implemented within Partial Least Square Discriminant Analysis (PLS-DA), describing the most discriminant species in descending order of importance. Each bar reports the following information: (i) length, VIP score; (ii) bar color, cohort in which the species has the highest mean relative abundance (high); (iii) edge color, cohort in which the species has the lowest mean relative abundance (low); iv) thickness, Fold Ratio (FR) among high and low; (v) significance of Mann-Whitney *U*-test among high and low (***P ≤ 0.001, ns, not significant). Mean relative abundance of microbiota at phylum, family, and species levels are reported as pie charts to provide a visual snapshot of the microbiota composition **(F)**.

**Figure 2:**
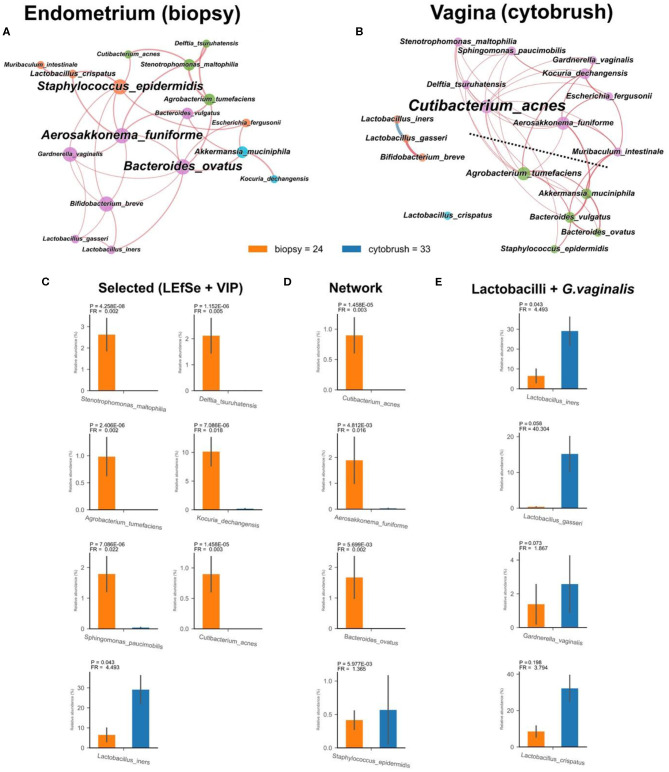
Endometrial (biopsy) and vaginal (cytobrush) tissues exhibit different bacterial networks with tissue-specific species. Co-occurrence bacterial networks were drawn for endometrial **(A)** and vaginal **(B)** microbiota. Each network was created by co-occurrence of 18 bacterial species having a prevalence ≥20% (the nodes) and concomitant significance of pair-wise Pearson correlation coefficient (the edges). Node properties are as follows: (i) size, normalized, and standardized bacterial relative abundances; (ii) color, communities as retrieved by Blondel algorithm; (iii) name size, betweenness centrality (a measure of the importance—the “keystoneness” within the network). Edge properties: (i) thickness, proportional to *P*-value of Pearson correlation coefficient divided into eight categories from the most significant (thicker) to the lesser one (thinner); (ii) color, red for positive, and blue for negative Pearson correlation coefficient. Dashed line, network “structural gap.” Relative abundances of selected species **(C)**, keystone species **(D)** and *Lactobacillus* spp. plus *G. vaginalis* **(E)** are reported as bar plots with SEM.

This pilot study enrolled 34 infertile women, divided into two groups according to the success or not of their ART cycle (Table 1). With the aim of establishing a possible difference between vaginal and uterine microbiota composition, we first compared 16S rRNA sequences from biopsies and from the cytobrush sampling of all patients without taking into account the success of pregnancy. Overall unsupervised Principal Component Analysis (PCoA, *P* = 9.9^*^10^−4^, ANOSIM = 0.281, Figure 1B), unsupervised Hierarchical Clustering Algorithm (HCA, Figure 1C) and supervised Partial Least Squares—Discriminant Analysis (PLS-DA, *P* = 9.9^*^10^−4^, ANOSIM = 0.835, not shown) revealed a significant difference between the two cohorts. Alfa-diversity also differed among biopsies and cytobrush samples, with fewer number of species and biodiversity within the vaginal microbial ecosystem (Figure 1A). In order to ascertain if specific bacterial species could be considered as potential biomarkers to discriminate vaginal and endometrial microbiota, we employed two algorithms: LEfSe (Figure 1D) and VIP (Figure 1E) with a two-stage Benjamini-Hochberg False Detection Rate (FDR) of 10%. Six biomarker species were ascribed to the endometrium (*Kocuria dechangensis, Sphingomonas paucimobilis, Stenotrophomonas maltophilia, Agrobacterium tumefaciens, Delftia tsuruhatensis, Cutibacterium acnes*, FR_average_ = 281.5, *P*adj_average_ = 2.82^*^10^−5^), while one biomarker species was ascribed to the vagina (*Lactobacillus iners*, FR = 4.5, *P* = 1.71^*^10^−2^) (Figures 1D,E). A visual snapshot of the microbiota composition in both tissues is shown in Figure 1F. Interestingly, within the endometrial network (Figure 2A) four of the six species belonged to a single community (green), while *L. iners* was significantly distinct (*P*adj = 6.5^*^10^−4^). However, this topological separation was not evident within the vaginal microbiota network (Figure 2B, *P* = 0.546). Other differences among the two bacterial networks are (Figure 2): (i) the endometrial microbiota has four linked communities, with a modularity equal to 0.36; (ii) the vaginal microbiota harbors two communities (purple and green) separated by a “structural gap” and a modularity equal to 0.38; (iii) the two species previously used to define the healthy (*L. crispatus*) or unhealthy (*L. iners*) status of the vaginal microenvironment are topologically not linked within the vagina (Figure 2B). However, within the endometrial microbiota these latter wpecies belong to two different communities (orange and purple), being separated by a minimum of two nodes (Figure 2A); (iv) *L. iners* and *L. gasseri* fall within the same community, but are positively related in the endometrium, while negatively related within the vagina. Of note, all *Lactobacillus* species of interest (*L. crispatus, L. gasseri, L. iners*—FR_average_ = 16.2), as well as *Gardnerella vaginalis* were more abundant in the vagina than the endometrium (Figure 2E), and this is further evident at the family level where Lactobacillaceae dominate the vaginal environment (Figure 1F). Through bacterial networks we were also able to identify keystone species (Figure 2D) within the endometrium (*Staphylococcus epidermidis, Aerosakkonema fusiforme, Bacteroides ovatus*) and vagina (*Cutibacterium acnes*), potentially seen as “topological biomarkers” for overall microbiota fitness within the two different habitats.

### Endometrial and Vaginal Microbial Community, Within Pregnant, and Non-pregnant Women

**Figure 3:**
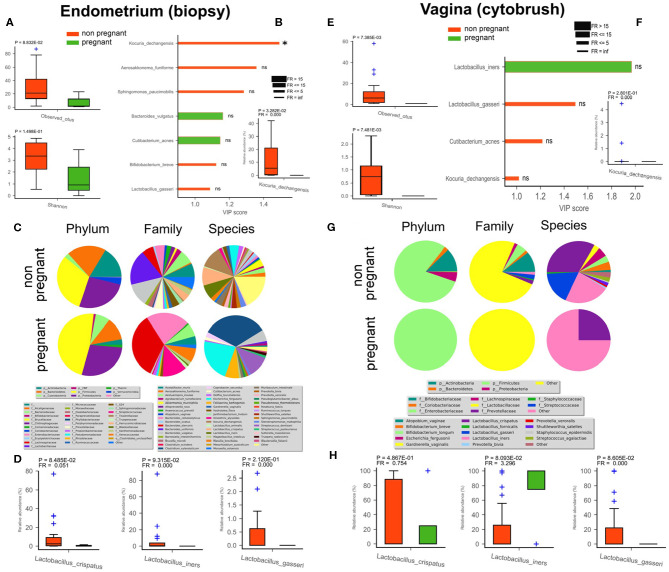
Differences in endometrial (biopsy) and vaginal (cytobrush) microbiota between pregnant (green) and non-pregnant (red) women. Alfa-diversity richness (observed OTUs) and biodiversity (Shannon) were assessed **(A,E)**. Biomarker species were found by PLS-DA VIP analysis **(B,F)**. Variable Importance Plot (VIP) was implemented within Partial Least Square Discriminant Analysis (PLS-DA), describing the most discriminant species in descending order of importance. Each bar reports the following information: (i) length, VIP score; (ii) bar color, cohort in which the species has the highest mean relative abundance (high); (iii) edge color, cohort in which the species has the lowest mean relative abundance (low); (iv) thickness, Fold Ratio (FR) among high and low; (v) significance of Mann-Whitney *U*-test among high and low (*P ≤ 0.05, ns = not significant). Mean relative */abundance of microbiota at phylum, family, and species levels are reported as pie charts to provide a visual snapshot of the general microbiota composition **(C,G)**. Relative abundance of three *Lactobacillus* spp. are reported as bar plots with SEM **(D,H)**.

In order to detect differences between pregnant and non-pregnant women in terms of microbiota composition (endometrium - Figures 3A–D; vagina - Figures 3E–H), we compared a group of four subjects who upon ART cycle became pregnant (4/34) with a second group composed of non-pregnant women irrespective of ART treatment (30/34). Among all the discriminant species within the non-pregnant group, evidenced by VIP plots, two were shared by the endometrium and vagina, namely *L. gasseri* and *Kocuria dechangensis*. *K. dechangensis* was the unique endometrial species significantly higher in relative abundance within non-pregnant women (*P* = 3.3^*^10^−2^, Figure 3B), while vaginal *L. gasseri* was significantly higher (*P* = 8.6^*^10^−2^, Figure 3H). These results highlighted a vaginal microbiota that seems to differ between the two groups. Principal Component Analysis PCoA and Partial least squares-discriminant analysis (PLSDA) applied to the uterus showed no significant difference among the endometrial microbiota of pregnant and non-pregnant women (data not shown). However, microbiota composition of endometrial and vaginal tissues appear to be different between the two groups studied (though our pregnant cohort was quite limited due to experimental constraints). The uterine bacterial community of non-pregnant women exhibited an interesting enrichment in *Lactobacilli* species normally present in the vaginal microbiota, and we also unveiled a bacterial biomarker species *Kocuria dechangensis*. The Gram+coccus bacterium Kocuria was identified as a common inhabitant of skin and oral mucosa (Grice et al., 2008). Recently, it was associated with urinary tract infection (Napolitani et al., 2019) and with infections in immunocompromised patients (Ma et al., 2005; Tsai et al., 2010). In line with these findings, we intriguingly observed that Kocuria specie was predominantly present in endometrial samples of non-pregnant women.

Next, the presence in the endometrial microbiota of non-pregnant women of species normally colonizing the vaginal district, likely arising coming from the vagina by translocation, could represent a negative factor related to the success of *in vitro* insemination and implantation. In our case, the vaginal flora of the four pregnant women was exclusively colonized by the Firmicutes phylum, with the Lactobacillaceae family being the main family within this phylum, whereas non-pregnant women displayed a more complex phylum spectrum (Figure 3G). At the species level only *L. iners, with* a significant predominance, and *L. crispatus* where present in pregnant group, while in non-pregnant women, *Gardnerella vaginalis* (phylum Actinobacteria), *Escherichia fergusonii* (phylum Proteobacteria), followed by *Streptococcus_dentisani* (phylum Firmicutes) seemed to be the predominant species. This datum was also corroborated by the alfa-diversity that showed a habitat richness and biodiversity significantly lower in the vagina of non-pregnant women. Overall, these preliminary results on vaginal and uterine microbiota indicate that changes in the composition appear to occur in pregnant woman, even though our cohort was composed of only four patients. Further studies with an extended cohort would thus be necessary to reinforce our findings. Interestingly, in the pregnant group a significant reduction of bacterial richness was observed in both the endometrium and vagina (Figures 3A,E), as well as fewer discriminant species (endometrium—*Bacteroides vulgatus, Cutibacterium acnes*; vagina—*L. iners*) (Figure 3F).

## Discussion

Previous studies focusing on the correlation between the microbiota and reproductive capacity of the woman, have highlighted the differences in vaginal microbiota between pregnant women and sterile women (van Oostrum et al., 2013; Romero et al., 2014; Sirota et al., 2014; Franasiak and Scott, 2015; Haahr et al., 2016; Wee et al., 2018). Moreover, the presence of species of the genus *Lactobacillus* is associated with a healthy genitourinary state, while a higher percentage of species such as *Gardnerella* and *Atopobium* is associated with dysbiosis, bacterial vaginosis, or inflammation (Moreno and Franasiak, 2017). Selman et al. examined the link between vaginal microbiota and infertility in patients undergoing *in vitro* fertilization (IVF). Positive patients for *Staphyloccocus* and Enterobacteriaceae showed lower implant rates compared to the group with more abundant Lactobacillus. However, the limit of the technique used makes this study incomplete (Selman et al., 2007). Van Oostrum et al. in their review, analyzed the correlation between bacterial vaginosis and the failure of IVF (van Oostrum et al., 2013).

Our preliminary results on vaginal microbiota seem to confirm previous literature. Indeed, in pregnant patients the microbiota appears to be dominated exclusively by two *Lactobacillus* species (*L. iners* and *L. crispatus*), while in non-pregnant women a richer vaginal ecosystem is present, along with a greater biodiversity. Though our cohorts were quite small to achieve reliable statistical and biological significance, it appears that the vaginal microbiota, in women who failed the implant after treatment, is a “non-lactobacillus dominant” (NLD) environment. Furthermore, in the group with failed implantation, we found *Kocuria dechangensis*, a gram positive and aerobic bacterium, which is now associated with urinary tract infection and is the cause of infections in immunocompromised patients and that could represent a bacterial biomarker indicative of possible implant failure. Several decades ago, the presence of bacteria in the uterine cavity was considered to be a risk factor for the woman and fetus (Martius and Eschenbach, 1990), but it was then shown that amniotic fluid, uterus, and placenta may instead accommodate unique microbiota (Aagaard et al., 2014; Franasiak and Scott, 2015; Collado et al., 2016). Currently, although still a matter of debate, the concept of “healthy” uterine microbiota has arisen, sustaining that Firmicutes, Bacteroidetes, Proteobacteria, and Actinobacteria represent the major phyla present. The presence of the phylum Firmicutes, in the case of the endometrial microbiota, remains controversial. Indeed, while some studies (Moreno and Simon, 2018) consider that *Lactobacillus* species are widely present in the uterus and constitute a marker of reproductive health, other articles relegate the presence of *Lactobacillus* to a vaginal contamination in sampling or even a pathophysiological condition of the uterus arising from the ascension of bacteria from the neighboring vagina (Baker et al., 2018). In Moreno's study, microbiota was characterized in endometrial fluid samples (more easily contaminated by the vaginal microbiota), not from biopsy. Similarly to the intestinal habitat, it is likely that the uterine mucosa-associated microbiota differs from that of the lumen. The validity of studies focusing on the uterine microbiota is affected by the high risk of uterine contamination by bacteria arising from the vagina and cervical canal. Franasiak et al., for example, investigated the bacterial flora at the time of IVF and embryo transfer and reported that Flavobacterium is one of the most abundant species in both types of patients, pregnant and non-pregnant (Khan et al., 2016). However, the presence of this bacterium within the uterine microbiota is not reported in an extensive number of articles in this field of research (Mitchell et al., 2015; Fang et al., 2016; Franasiak et al., 2016; Khan et al., 2016; Moreno et al., 2016; Verstraelen et al., 2016; Walther-António et al., 2016; Chen et al., 2017; Miles et al., 2017; Tao et al., 2017), but seems to be related to a water contamination or to the kit used for the study (the Ion metagenomics kit).

Excluding studies by Miles et al. and Walther-António et al., in which the uterine biopsies were acquired after hysterectomy (Walther-António et al., 2016; Miles et al., 2017) in our pilot study we can be reasonably sure that the samples were obtained without contamination between the lower (vagina) genital tract, and cervical canal (uterine cavity). The technique we used to obtain endometrial material is the biopsy of the tissue. This material is more representative of the uterine cavity microbiota (Fang et al., 2016; Verstraelen et al., 2016; Chen et al., 2017; Miles et al., 2017; Liu et al., 2018) in comparison with the other studies that used only uterine fluid extracted by endometrial swab or embryo-transfer catheter tip analysis (Franasiak et al., 2016; Khan et al., 2016; Moreno et al., 2016; Tao et al., 2017; Kyono et al., 2018, 2019; Pelzer et al., 2018).

In a recent study on 25 women, Liu at al collected both endometrial fluid and tissue to compare their microbiota composition. While the extraction of endometrial fluid was conducted without contamination between the lower genital tract and the uterine cavity by using the embryo-transfer catheter protection, the tissue biopsy was obtained by using the Pipelle catheter, resulting in the potential contamination by bacteria from the vagina and cervical canal (Liu et al., 2018). Moreover, in order to obtain a complete characterization and more representative composition of the uterine microbiota, we avoided using lower genital tract disinfection and pre-procedural antibiotics, unlike several previous studies (Fang et al., 2016; Miles et al., 2017; Wee et al., 2018).

The results obtained in this pilot study show differences in microbiota composition between the vaginal and uterine habitat. The *Lactobacillus* genus represents the dominant genera in the vaginal microbiota, while the uterine microbiota is characterized by a more heterogeneous composition with the presence of species as yet undetected before in human genital areas, such as *Kocuria dechangensis*. This significant difference, along with an almost total absence of *Lactobacillus* genus in the uterine microbiota, excludes any contamination from the neighboring vagina at the time of sampling, and highlights a different composition of the endometrial microbiota. A clear difference in mucosa endometrial microbiota between pregnant and non-pregnant women was also revealed in our study. The pregnant group, compared to the non-pregnant group, was characterized by the total absence of the *Lactobacillus* genus and the predominant presence of Lachnospiraceae and Enterobacteriaceae with a poorer alpha-diversity. The endometrial microbiota of non-pregnant women displayed an increase in *Lactobacillus* species, that could have arisen from the vaginal. *Lactobacillus* translocation may be due to a malfunction of barriers that normally prevent this shifting. The colonization by *Lactobacillus* species, at the level of the endometrial mucosa, could have shaped an unfavorable habitat for IVF outcome. Among the four pregnant women, two experienced intrauterine hysteroscopy (one had operative hysteroscopy for U3 uterine anomaly; and one had hysteroscopy for G1 fibroid) performed at least 12 months prior to the ART cycle. Since it is well-known that an increase in pregnancy outcome is achieved in the 3–6 months following hysteroscopy, we would like to underline that in our patients a correlation between the increase in pregnancy outcome and hysteroscopy was thus avoided (Neerja and Jain, 2014; Di Spiezio Sardo et al., 2016; Piccioni et al., 2017; Riganelli et al., 2018).

We are aware that the preliminary results discussed in this pilot study need to be extended. However, the results obtained represent the first indication, from sequencing data, that microbiota mucosa associated *in utero* is structurally different from the vaginal one. It seems that when a translocation from the vagina to the endometrial area happens, probably due to the reduction of barriers, this may lead to an unsuitable microbiota, which may negatively affect IVF result. Moreover, results suggest that the evaluation of a predictive “microbiota dysbiosis,” before an ART treatment is advantageous. These preliminary results argue in favor of restoring the vaginal and/or endometrial microenvironment, by the combined use of hormonal stimulation and targeted probiotic and/or antibiotic therapies, to improve IVF outcome. The prior evaluation of the microbiota composition (vaginal and endometrial) in women who need to undergo IVF could enable clinicians to restore a balanced microbiota, and to administer personalized therapies.

## Data Availability Statement

Raw data (fastq.gz files) are available at the National Center for Biotechnology Information (NCBI) Sequence Read Archive (SRA) database under the Bioproject PRJNA603234.

## Ethics Statement

The study has been conducted according to the ethical standards expressed in the Declaration of Helsinki. It has been approved by the University of Rome “Sapienza” ethics committee (protocol number: AR11715C81FB56F4) and all subjects have provided written informed consent.

## Author Contributions

FG, SS, LR, and MP conceived and designed the study. LR, LM, MM, and DP collected samples and clinical data. FG, ML, II, MM, GB, BN, LC, and AG performed experiments. VI performed bioinformatics analysis. LM, DP, and OC contributed to the discussion. FG, SS, VI, LR, and DP interpreted data. FG, SS, VI, and LR wrote the paper. All authors contributed to the article and approved the submitted version.

## Conflict of Interest

The authors declare that the research was conducted in the absence of any commercial or financial relationships that could be construed as a potential conflict of interest.
