# Supplementary material for: Structural Variations of Vaginal and Endometrial Microbiota: Hints on Female Infertility
Source: Front Cell Infect Microbiol. 2020 Jul 14;10:350. doi: 10.3389/fcimb.2020.00350 (PMC7372811; doi:10.3389/fcimb.2020.00350)
Supplement: Supplementary file 1 [file Data_Sheet_1.PDF]

SUPPLEMENTARY MATERIAL

Structural variations of vaginal and endometrial microbiota: hints on female infertility

Riganelli L*, Iebba V*, Piccioni MG, Illuminati I, Bonfiglio G, Neroni B, Calvo L, Gagliardi A, Levrero M, Merlino L, Mariani M, Capri O, Pietrangeli D, Schippa S**°, Guerrieri F**°

Supplementary Figure 1. Theoretical distribution of the 8 bacterial species available as a "mock" community within the ZymoBIOMICS Microbial Community DNA Standard D6305 was compared with the distribution obtained with our MiSeq sequencing platform taking into consideration two different Taq polymerases.

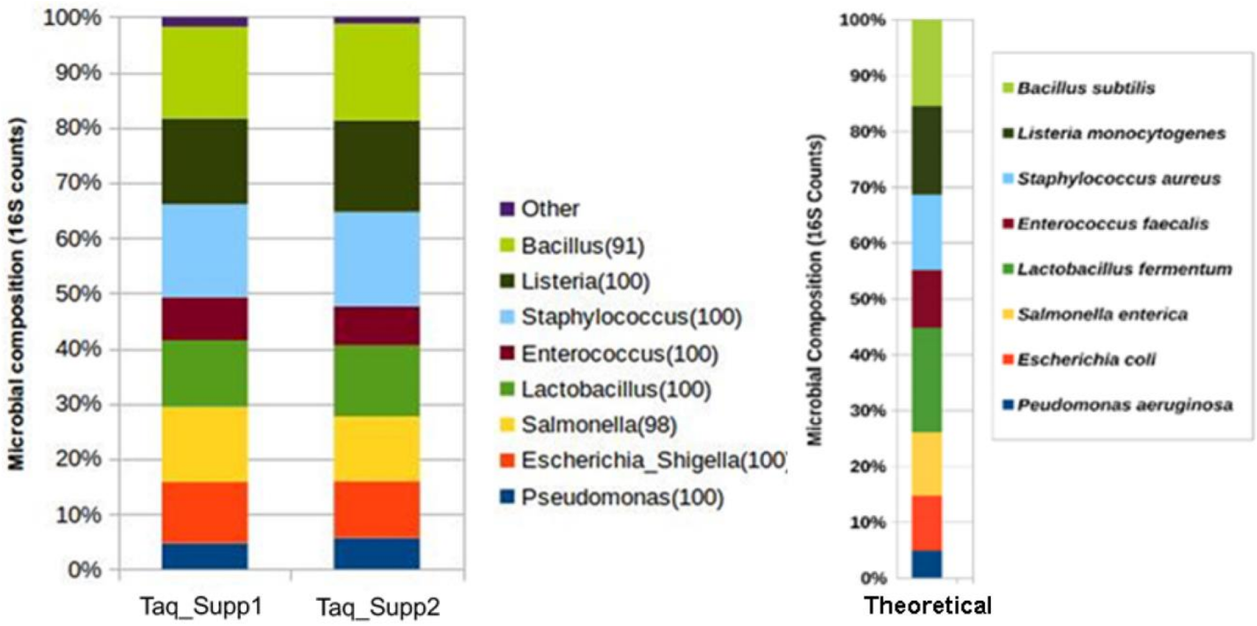

Supplementary Text 1 (following pages). Mothur v1.39.5 pipeline was used to analyze raw fastq files. All steps are presented here as they were implemented following a modified version of the MiSeq SOP (https://mothur.org/wiki/miseq_sop/).

Mothur.txt

```
Linux version

Using ReadLine

Running 64Bit Version

mothur v.1.39.5
Last updated: 3/20/2017

by
Patrick D. Schloss

Department of Microbiology & Immunology
University of Michigan
http://www.mothur.org

When using, please cite:
Schloss, P.D., et al., Introducing mothur: Open-source, platform-independent, community-supported
software for describing and comparing microbial communities. Appl Environ Microbiol, 2009.
75(23):7537-41.

Distributed under the GNU General Public License

Type 'help()' for information on the commands that are available

For questions and analysis support, please visit our forum at https://www.mothur.org/forum

Type 'quit()' to exit program
Batch Mode


mothur > make.contigs(file=stability.files, processors=24)

Using 24 processors.

>>>>> Processing file pair 35G_S64_L001_R1_001.fastq.gz - 35G_S64_L001_R2_001.fastq.gz (files 21 of
68) <<<<<
Making contigs...
Done.

It took 12 secs to assemble 5516 reads.


mothur > make.contigs(file=stability.files, processors=24)

Using 24 processors.

>>>>> Processing file pair 65G_S3_L001_R1_001.fastq.gz - 65G_S3_L001_R2_001.fastq.gz (files 57 of
68) <<<<<
Making contigs...
Done.

It took 19 secs to assemble 8071 reads.


mothur > make.contigs(file=stability.files, processors=24)

Using 24 processors.

>>>>> Processing file pair 13b_S25_L001_R1_001.fastq.gz - 13b_S25_L001_R2_001.fastq.gz (files 7 of
68) <<<<<
Making contigs...
Done.

It took 60 secs to assemble 25756 reads.
```

```
mothur > make.contigs(file=stability.files, processors=24)

Using 24 processors.

>>>>> Processing file pair 45G_S74_L001_R1_001.fastq.gz - 45G_S74_L001_R2_001.fastq.gz (files 33 of
68) <<<<<
Making contigs...
Done.

It took 72 secs to assemble 28775 reads.


mothur > make.contigs(file=stability.files, processors=24)

Using 24 processors.

>>>>> Processing file pair 14b_S27_L001_R1_001.fastq.gz - 14b_S27_L001_R2_001.fastq.gz (files 9 of
68) <<<<<
Making contigs...
Done.

It took 81 secs to assemble 34727 reads.


mothur > make.contigs(file=stability.files, processors=24)

Using 24 processors.

>>>>> Processing file pair 32G_S61_L001_R1_001.fastq.gz - 32G_S61_L001_R2_001.fastq.gz (files 18 of
68) <<<<<
Making contigs...
Done.

It took 82 secs to assemble 32602 reads.


>>>>> Processing file pair 33G_S62_L001_R1_001.fastq.gz - 33G_S62_L001_R2_001.fastq.gz (files 19 of
68) <<<<<
Making contigs...
Done.

It took 11 secs to assemble 4491 reads.


mothur > make.contigs(file=stability.files, processors=24)

Using 24 processors.

>>>>> Processing file pair 58G_S87_L001_R1_001.fastq.gz - 58G_S87_L001_R2_001.fastq.gz (files 48 of
68) <<<<<
Making contigs...
Done.

It took 93 secs to assemble 41133 reads.


mothur > make.contigs(file=stability.files, processors=24)

Using 24 processors.

>>>>> Processing file pair 15c_S30_L001_R1_001.fastq.gz - 15c_S30_L001_R2_001.fastq.gz (files 12 of
68) <<<<<
Making contigs...
Done.

It took 96 secs to assemble 36241 reads.
```

```
>>>>> Processing file pair 34G_S63_L001_R1_001.fastq.gz - 34G_S63_L001_R2_001.fastq.gz (files 20 of
68) <<<<<
Making contigs...
Done.

It took 6 secs to assemble 2619 reads.


mothur > make.contigs(file=stability.files, processors=24)

Using 24 processors.

>>>>> Processing file pair 7b_S13_L001_R1_001.fastq.gz - 7b_S13_L001_R2_001.fastq.gz (files 63 of
68) <<<<<
Making contigs...
Done.

It took 106 secs to assemble 43368 reads.


mothur > make.contigs(file=stability.files, processors=24)

Using 24 processors.

>>>>> Processing file pair 12b_S23_L001_R1_001.fastq.gz - 12b_S23_L001_R2_001.fastq.gz (files 5 of
68) <<<<<
Making contigs...
Done.

It took 122 secs to assemble 55968 reads.


mothur > make.contigs(file=stability.files, processors=24)

Using 24 processors.

>>>>> Processing file pair 38G_S67_L001_R1_001.fastq.gz - 38G_S67_L001_R2_001.fastq.gz (files 24 of
68) <<<<<
Making contigs...
Done.

It took 124 secs to assemble 47184 reads.


>>>>> Processing file pair 36G_S65_L001_R1_001.fastq.gz - 36G_S65_L001_R2_001.fastq.gz (files 22 of
68) <<<<<
Making contigs...
Done.

It took 126 secs to assemble 46372 reads.


>>>>> Processing file pair 37G_S66_L001_R1_001.fastq.gz - 37G_S66_L001_R2_001.fastq.gz (files 23 of
68) <<<<<
Making contigs...
Done.

It took 19 secs to assemble 8167 reads.


mothur > make.contigs(file=stability.files, processors=24)

Using 24 processors.

>>>>> Processing file pair 10b_S19_L001_R1_001.fastq.gz - 10b_S19_L001_R2_001.fastq.gz (files 1 of
68) <<<<<
```

```
Making contigs...
Done.

It took 165 secs to assemble 66533 reads.


>>>>> Processing file pair 66G_S4_L001_R1_001.fastq.gz - 66G_S4_L001_R2_001.fastq.gz (files 58 of
68) <<<<<
Making contigs...
Done.

It took 148 secs to assemble 55852 reads.


>>>>> Processing file pair 7c_S14_L001_R1_001.fastq.gz - 7c_S14_L001_R2_001.fastq.gz (files 64 of
68) <<<<<
Making contigs...
Done.

It took 67 secs to assemble 27149 reads.


>>>>> Processing file pair 39G_S68_L001_R1_001.fastq.gz - 39G_S68_L001_R2_001.fastq.gz (files 25 of
68) <<<<<
Making contigs...
Done.

It took 56 secs to assemble 23215 reads.


mothur > make.contigs(file=stability.files, processors=24)

Using 24 processors.

>>>>> Processing file pair 42G_S71_L001_R1_001.fastq.gz - 42G_S71_L001_R2_001.fastq.gz (files 30 of
68) <<<<<
Making contigs...
Done.

It took 190 secs to assemble 70918 reads.


mothur > make.contigs(file=stability.files, processors=24)

Using 24 processors.

>>>>> Processing file pair 11b_S21_L001_R1_001.fastq.gz - 11b_S21_L001_R2_001.fastq.gz (files 3 of
68) <<<<<
Making contigs...
Done.

It took 195 secs to assemble 84520 reads.


>>>>> Processing file pair 1b_S1_L001_R1_001.fastq.gz - 1b_S1_L001_R2_001.fastq.gz (files 13 of 68)
<<<<<
Making contigs...
Done.

It took 109 secs to assemble 48893 reads.


>>>>> Processing file pair 59G_S88_L001_R1_001.fastq.gz - 59G_S88_L001_R2_001.fastq.gz (files 49 of
68) <<<<<
Making contigs...
Done.

It took 120 secs to assemble 50465 reads.
```

```
>>>>> Processing file pair 67G_S5_L001_R1_001.fastq.gz - 67G_S5_L001_R2_001.fastq.gz (files 59 of
68) <<<<<
Making contigs...
Done.

It took 53 secs to assemble 22386 reads.


>>>>> Processing file pair 43G_S72_L001_R1_001.fastq.gz - 43G_S72_L001_R2_001.fastq.gz (files 31 of
68) <<<<<
Making contigs...
Done.

It took 36 secs to assemble 15288 reads.


>>>>> Processing file pair 14c_S28_L001_R1_001.fastq.gz - 14c_S28_L001_R2_001.fastq.gz (files 10 of
68) <<<<<
Making contigs...
Done.

It took 164 secs to assemble 62883 reads.


>>>>> Processing file pair 8b_S15_L001_R1_001.fastq.gz - 8b_S15_L001_R2_001.fastq.gz (files 65 of
68) <<<<<
Making contigs...
Done.

It took 73 secs to assemble 33352 reads.


>>>>> Processing file pair 5b_S9_L001_R1_001.fastq.gz - 5b_S9_L001_R2_001.fastq.gz (files 50 of 68)
<<<<<
Making contigs...
Done.

It took 48 secs to assemble 20702 reads.


mothur > make.contigs(file=stability.files, processors=24)

Using 24 processors.

>>>>> Processing file pair 2b_S3_L001_R1_001.fastq.gz - 2b_S3_L001_R2_001.fastq.gz (files 15 of 68)
<<<<<
Making contigs...
Done.

It took 264 secs to assemble 110830 reads.


mothur > make.contigs(file=stability.files, processors=24)

Using 24 processors.

>>>>> Processing file pair 8c_S16_L001_R1_001.fastq.gz - 8c_S16_L001_R2_001.fastq.gz (files 66 of
68) <<<<<
Making contigs...
Done.

It took 267 secs to assemble 103001 reads.


>>>>> Processing file pair 15b_S29_L001_R1_001.fastq.gz - 15b_S29_L001_R2_001.fastq.gz (files 11 of
68) <<<<<
```

```
Making contigs...
Done.

It took 26 secs to assemble 11697 reads.


>>>>> Processing file pair 3b_S5_L001_R1_001.fastq.gz - 3b_S5_L001_R2_001.fastq.gz (files 26 of 68)
<<<<<
Making contigs...
Done.

It took 154 secs to assemble 78508 reads.


mothur > make.contigs(file=stability.files, processors=24)

Using 24 processors.

>>>>> Processing file pair 55G_S84_L001_R1_001.fastq.gz - 55G_S84_L001_R2_001.fastq.gz (files 45 of
68) <<<<<
Making contigs...
Done.

It took 338 secs to assemble 133693 reads.


mothur > make.contigs(file=stability.files, processors=24)

Using 24 processors.

>>>>> Processing file pair 5c_S10_L001_R1_001.fastq.gz - 5c_S10_L001_R2_001.fastq.gz (files 51 of
68) <<<<<
Making contigs...
Done.

It took 348 secs to assemble 139259 reads.


>>>>> Processing file pair 9b_S17_L001_R1_001.fastq.gz - 9b_S17_L001_R2_001.fastq.gz (files 67 of
68) <<<<<
Making contigs...
Done.

It took 104 secs to assemble 58025 reads.


>>>>> Processing file pair 10c_S20_L001_R1_001.fastq.gz - 10c_S20_L001_R2_001.fastq.gz (files 2 of
68) <<<<<
Making contigs...
Done.

It took 209 secs to assemble 88987 reads.


mothur > make.contigs(file=stability.files, processors=24)

Using 24 processors.

>>>>> Processing file pair 52G_S81_L001_R1_001.fastq.gz - 52G_S81_L001_R2_001.fastq.gz (files 42 of
68) <<<<<
Making contigs...
Done.

It took 395 secs to assemble 160900 reads.


mothur > make.contigs(file=stability.files, processors=24)
```

Using 24 processors.

>>>>> Processing file pair 68G_S6_L001_R1_001.fastq.gz - 68G_S6_L001_R2_001.fastq.gz (files 60 of 68) <<<<<
Making contigs...
Done.

It took 405 secs to assemble 163428 reads.

>>>>> Processing file pair 46G_S75_L001_R1_001.fastq.gz - 46G_S75_L001_R2_001.fastq.gz (files 34 of 68) <<<<<
Making contigs...
Done.

It took 335 secs to assemble 135146 reads.

>>>>> Processing file pair 2c_S4_L001_R1_001.fastq.gz - 2c_S4_L001_R2_001.fastq.gz (files 16 of 68) <<<<<
Making contigs...
Done.

It took 155 secs to assemble 72440 reads.

>>>>> Processing file pair 31G_S60_L001_R1_001.fastq.gz - 31G_S60_L001_R2_001.fastq.gz (files 17 of 68) <<<<<
Making contigs...
Done.

It took 4 secs to assemble 2408 reads.

>>>>> Processing file pair 53G_S82_L001_R1_001.fastq.gz - 53G_S82_L001_R2_001.fastq.gz (files 43 of 68) <<<<<
Making contigs...
Done.

It took 49 secs to assemble 26431 reads.

mothur > make.contigs(file=stability.files, processors=24)

Using 24 processors.

>>>>> Processing file pair 3c_S6_L001_R1_001.fastq.gz - 3c_S6_L001_R2_001.fastq.gz (files 27 of 68) <<<<<
Making contigs...
Done.

It took 447 secs to assemble 184526 reads.

>>>>> Processing file pair 47G_S76_L001_R1_001.fastq.gz - 47G_S76_L001_R2_001.fastq.gz (files 35 of 68) <<<<<
Making contigs...
Done.

It took 47 secs to assemble 26167 reads.

mothur > make.contigs(file=stability.files, processors=24)

Using 24 processors.

>>>>> Processing file pair 4c_S8_L001_R1_001.fastq.gz - 4c_S8_L001_R2_001.fastq.gz (files 39 of 68) <<<<<

```
Making contigs...
Done.

It took 455 secs to assemble 184796 reads.


>>>>> Processing file pair 6b_S11_L001_R1_001.fastq.gz - 6b_S11_L001_R2_001.fastq.gz (files 61 of
68) <<<<<
Making contigs...
Done.

It took 50 secs to assemble 24638 reads.


mothur > make.contigs(file=stability.files, processors=24)

Using 24 processors.

>>>>> Processing file pair 48G_S77_L001_R1_001.fastq.gz - 48G_S77_L001_R2_001.fastq.gz (files 36 of
68) <<<<<
Making contigs...
Done.

It took 458 secs to assemble 185701 reads.


>>>>> Processing file pair 6c_S12_L001_R1_001.fastq.gz - 6c_S12_L001_R2_001.fastq.gz (files 62 of
68) <<<<<
Making contigs...
Done.

It took 100 secs to assemble 33081 reads.


>>>>> Processing file pair 13c_S26_L001_R1_001.fastq.gz - 13c_S26_L001_R2_001.fastq.gz (files 8 of
68) <<<<<
Making contigs...
Done.

It took 502 secs to assemble 204433 reads.


>>>>> Processing file pair 44G_S73_L001_R1_001.fastq.gz - 44G_S73_L001_R2_001.fastq.gz (files 32 of
68) <<<<<
Making contigs...
Done.

It took 345 secs to assemble 143984 reads.


mothur > make.contigs(file=stability.files, processors=24)

Using 24 processors.

>>>>> Processing file pair 62G_S91_L001_R1_001.fastq.gz - 62G_S91_L001_R2_001.fastq.gz (files 54 of
68) <<<<<
Making contigs...
Done.

It took 607 secs to assemble 253223 reads.


>>>>> Processing file pair 49G_S78_L001_R1_001.fastq.gz - 49G_S78_L001_R2_001.fastq.gz (files 37 of
68) <<<<<
Making contigs...
Done.

It took 152 secs to assemble 66593 reads.
```

```
>>>>> Processing file pair 63G_S1_L001_R1_001.fastq.gz - 63G_S1_L001_R2_001.fastq.gz (files 55 of
68) <<<<<
Making contigs...
Done.

It took 13 secs to assemble 8405 reads.


>>>>> Processing file pair 1c_S2_L001_R1_001.fastq.gz - 1c_S2_L001_R2_001.fastq.gz (files 14 of 68)
<<<<<
Making contigs...
Done.

It took 491 secs to assemble 217265 reads.


>>>>> Processing file pair 50G_S79_L001_R1_001.fastq.gz - 50G_S79_L001_R2_001.fastq.gz (files 40 of
68) <<<<<
Making contigs...
Done.

It took 242 secs to assemble 111822 reads.


>>>>> Processing file pair 4b_S7_L001_R1_001.fastq.gz - 4b_S7_L001_R2_001.fastq.gz (files 38 of 68)
<<<<<
Making contigs...
Done.

It took 91 secs to assemble 59763 reads.


>>>>> Processing file pair 40G_S69_L001_R1_001.fastq.gz - 40G_S69_L001_R2_001.fastq.gz (files 28 of
68) <<<<<
Making contigs...
Done.

It took 257 secs to assemble 124223 reads.


>>>>> Processing file pair 11c_S22_L001_R1_001.fastq.gz - 11c_S22_L001_R2_001.fastq.gz (files 4 of
68) <<<<<
Making contigs...
Done.

It took 524 secs to assemble 235710 reads.


>>>>> Processing file pair 56G_S85_L001_R1_001.fastq.gz - 56G_S85_L001_R2_001.fastq.gz (files 46 of
68) <<<<<
Making contigs...
Done.

It took 384 secs to assemble 180501 reads.


>>>>> Processing file pair 41G_S70_L001_R1_001.fastq.gz - 41G_S70_L001_R2_001.fastq.gz (files 29 of
68) <<<<<
Making contigs...
Done.

It took 21 secs to assemble 14468 reads.


>>>>> Processing file pair 51G_S80_L001_R1_001.fastq.gz - 51G_S80_L001_R2_001.fastq.gz (files 41 of
68) <<<<<
```

```
Making contigs...
Done.

It took 37 secs to assemble 28930 reads.


>>>>> Processing file pair 12c_S24_L001_R1_001.fastq.gz - 12c_S24_L001_R2_001.fastq.gz (files 6 of
68) <<<<<
Making contigs...
Done.

It took 658 secs to assemble 305319 reads.


>>>>> Processing file pair 60G_S89_L001_R1_001.fastq.gz - 60G_S89_L001_R2_001.fastq.gz (files 52 of
68) <<<<<
Making contigs...
Done.

It took 434 secs to assemble 219148 reads.


>>>>> Processing file pair 57G_S86_L001_R1_001.fastq.gz - 57G_S86_L001_R2_001.fastq.gz (files 47 of
68) <<<<<
Making contigs...
Done.

It took 99 secs to assemble 79546 reads.


>>>>> Processing file pair 61G_S90_L001_R1_001.fastq.gz - 61G_S90_L001_R2_001.fastq.gz (files 53 of
68) <<<<<
Making contigs...
Done.

It took 54 secs to assemble 46170 reads.


>>>>> Processing file pair 54G_S83_L001_R1_001.fastq.gz - 54G_S83_L001_R2_001.fastq.gz (files 44 of
68) <<<<<
Making contigs...
Done.

It took 399 secs to assemble 214078 reads.


>>>>> Processing file pair 64G_S2_L001_R1_001.fastq.gz - 64G_S2_L001_R2_001.fastq.gz (files 56 of
68) <<<<<
Making contigs...
Done.

It took 320 secs to assemble 206065 reads.


>>>>> Processing file pair 9c_S18_L001_R1_001.fastq.gz - 9c_S18_L001_R2_001.fastq.gz (files 68 of
68) <<<<<
Making contigs...
Done.

It took 607 secs to assemble 349913 reads.

It took 1022 secs to process 5996367 sequences.

Group count:
10b 66533
10c 88987
11b 84520
11c 235710
```

```
12b 55968
12c 305319
13b 25756
13c 204433
14b 34727
14c 62883
15b 11697
15c 36241
1b 48893
1c 217265
2b 110830
2c 72440
31G 2408
32G 32602
33G 4491
34G 2619
35G 5516
36G 46372
37G 8167
38G 47184
39G 23215
3b 78508
3c 184526
40G 124223
41G 14468
42G 70918
43G 15288
44G 143984
45G 28775
46G 135146
47G 26167
48G 185701
49G 66593
4b 59763
4c 184796
50G 111822
51G 28930
52G 160900
53G 26431
54G 214078
55G 133693
56G 180501
57G 79546
58G 41133
59G 50465
5b 20702
5c 139259
60G 219148
61G 46170
62G 253223
63G 8405
64G 206065
65G 8071
66G 55852
67G 22386
68G 163428
6b 24638
6c 33081
7b 43368
7c 27149
8b 33352
8c 103001
9b 58025
9c 349913

Total of all groups is 5996367

Output File Names:
```

```
stability.trim.contigs.fasta
stability.trim.contigs.qual
stability.contigs.report
stability.scrap.contigs.fasta
stability.scrap.contigs.qual
stability.contigs.groups

[WARNING]: your sequence names contained ':'. I changed them to '_' to avoid problems in your
downstream analysis.

mothur > summary.seqs(fasta=stability.trim.contigs.fasta, processors=24)

Using 24 processors.

Start End NBases Ambigs Polymer NumSeqs
Minimum: 1 35 35 0 2 1
2.5%-tile: 1 290 290 0 3 149910
25%-tile: 1 450 450 0 4 1499092
Median: 1 465 465 3 5 2998184
75%-tile: 1 465 465 10 6 4497276
97.5%-tile: 1 598 598 27 13 5846458
Maximum: 1 602 602 271 300 5996367
Mean: 1 452.888 452.888 6.50191 5.57219
# of Seqs: 5996367

Output File Names:
stability.trim.contigs.summary

It took 10 secs to summarize 5996367 sequences.

mothur > screen.seqs(fasta=stability.trim.contigs.fasta, group=stability.contigs.groups, maxambig=0,
maxhomop=9, minlength=400, maxlength=500)

Using 24 processors.

Output File Names:
stability.trim.contigs.good.fasta
stability.trim.contigs.bad.accnos
stability.contigs.good.groups


It took 32 secs to screen 5996367 sequences.

mothur > summary.seqs(fasta=stability.trim.contigs.good.fasta)

Using 24 processors.

Start End NBases Ambigs Polymer NumSeqs
Minimum: 1 400 400 0 3 1
2.5%-tile: 1 439 439 0 3 27474
25%-tile: 1 465 465 0 4 274735
Median: 1 465 465 0 5 549469
75%-tile: 1 465 465 0 5 824203
97.5%-tile: 1 465 465 0 6 1071464
Maximum: 1 500 500 0 9 1098937
Mean: 1 461.118 461.118 0 4.7133
# of Seqs: 1098937

Output File Names:
stability.trim.contigs.good.summary

It took 2 secs to summarize 1098937 sequences.

mothur > unique.seqs(fasta=stability.trim.contigs.good.fasta)
1098937 434720

Output File Names:
stability.trim.contigs.good.names
```

```
stability.trim.contigs.good.unique.fasta


mothur > count.seqs(name=stability.trim.contigs.good.names, group=stability.contigs.good.groups)

Using 24 processors.
It took 4 secs to create a table for 1098937 sequences.


Total number of sequences: 1098937

Output File Names:
stability.trim.contigs.good.count_table


mothur > summary.seqs(count=stability.trim.contigs.good.count_table)
Using stability.trim.contigs.good.unique.fasta as input file for the fasta parameter.

Using 24 processors.

Start End NBases Ambigs Polymer NumSeqs
Minimum: 1 400 400 0 3 1
2.5%-tile: 1 439 439 0 3 27474
25%-tile: 1 465 465 0 4 274735
Median: 1 465 465 0 5 549469
75%-tile: 1 465 465 0 5 824203
97.5%-tile: 1 465 465 0 6 1071464
Maximum: 1 500 500 0 9 1098937
Mean: 1 461.118 461.118 0 4.7133
# of unique seqs: 434720
total # of seqs: 1098937

Output File Names:
stability.trim.contigs.good.unique.summary

It took 3 secs to summarize 1098937 sequences.

mothur > align.seqs(fasta=stability.trim.contigs.good.unique.fasta, reference=silva.v3v4.fasta,
processors=24)

Using 24 processors.

Reading in the silva.v3v4.fasta template sequences... DONE.
It took 6 to read 14956 sequences.
Aligning sequences from stability.trim.contigs.good.unique.fasta ...
[WARNING]: Some of your sequences generated alignments that eliminated too many bases, a list is
provided in stability.trim.contigs.good.unique.flip.accnos. If you set the flip parameter to true
mothur will try aligning the reverse compliment as well.
It took 203 secs to align 434720 sequences.


Output File Names:
stability.trim.contigs.good.unique.align
stability.trim.contigs.good.unique.align.report
stability.trim.contigs.good.unique.flip.accnos


mothur > summary.seqs(fasta=stability.trim.contigs.good.unique.align,
count=stability.trim.contigs.good.count_table)

Using 24 processors.

Start End NBases Ambigs Polymer NumSeqs
Minimum: 0 0 0 0 1 1
2.5%-tile: 1 18928 12 0 3 27474
25%-tile: 1 18928 464 0 4 274735
Median: 1 18928 464 0 5 549469
75%-tile: 1 18928 464 0 5 824203
```

```
97.5%-tile: 1 18928 464 0 6 1071464
Maximum: 18928 18928 469 0 9 1098937
Mean: 455.032 18873.4 448.427 0 4.58905
# of unique seqs: 434720
total # of seqs: 1098937

Output File Names:
stability.trim.contigs.good.unique.summary

It took 19 secs to summarize 1098937 sequences.

mothur > screen.seqs(fasta=stability.trim.contigs.good.unique.align, count=current, summary=current,
maxambig=0, maxhomop=9, start=1, end=18928, minlength=400, maxlength=500, processors=24)
Using stability.trim.contigs.good.count_table as input file for the count parameter.
Using stability.trim.contigs.good.unique.summary as input file for the summary parameter.

Using 24 processors.

Output File Names:
stability.trim.contigs.good.unique.good.summary
stability.trim.contigs.good.unique.good.align
stability.trim.contigs.good.unique.bad.accnos
stability.trim.contigs.good.good.count_table


It took 55 secs to screen 434720 sequences.

mothur > summary.seqs(fasta=current, count=current)
Using stability.trim.contigs.good.good.count_table as input file for the count parameter.
Using stability.trim.contigs.good.unique.good.align as input file for the fasta parameter.

Using 24 processors.

Start End NBases Ambigs Polymer NumSeqs
Minimum: 1 18928 434 0 3 1
2.5%-tile: 1 18928 441 0 3 26653
25%-tile: 1 18928 464 0 4 266521
Median: 1 18928 464 0 5 533042
75%-tile: 1 18928 464 0 5 799562
97.5%-tile: 1 18928 464 0 6 1039430
Maximum: 1 18928 469 0 9 1066082
Mean: 1 18928 460.779 0 4.67756
# of unique seqs: 406473
total # of seqs: 1066082

Output File Names:
stability.trim.contigs.good.unique.good.summary

It took 19 secs to summarize 1066082 sequences.

mothur > filter.seqs(fasta=stability.trim.contigs.good.unique.good.align, vertical=T, trump=.)

Using 24 processors.
Creating Filter...


Running Filter...



Length of filtered alignment: 890
Number of columns removed: 18038
Length of the original alignment: 18928
Number of sequences used to construct filter: 406473

Output File Names:
stability.filter
stability.trim.contigs.good.unique.good.filter.fasta
```

```
mothur > unique.seqs(fasta=stability.trim.contigs.good.unique.good.filter.fasta,
count=stability.trim.contigs.good.good.count_table)
406473 402141

Output File Names:
stability.trim.contigs.good.unique.good.filter.count_table
stability.trim.contigs.good.unique.good.filter.unique.fasta


mothur > pre.cluster(fasta=current, count=current, diffs=5, processors=24)
Using stability.trim.contigs.good.unique.good.filter.count_table as input file for the count
parameter.
Using stability.trim.contigs.good.unique.good.filter.unique.fasta as input file for the fasta
parameter.

Using 24 processors.

Processing group 7b:
11 6 5
Total number of sequences before pre.cluster was 11.
pre.cluster removed 5 sequences.

It took 0 secs to cluster 11 sequences.

Processing group 7c:
1 1 0
Total number of sequences before pre.cluster was 1.
pre.cluster removed 0 sequences.

It took 0 secs to cluster 1 sequences.

Processing group 8b:
8 7 1
Total number of sequences before pre.cluster was 8.
pre.cluster removed 1 sequences.

It took 0 secs to cluster 8 sequences.

Using 24 processors.

Processing group 5c:
1773 355 1418
Total number of sequences before pre.cluster was 1773.
pre.cluster removed 1418 sequences.

It took 0 secs to cluster 1773 sequences.

Processing group 60G:

Using 24 processors.

Processing group 48G:

Using 24 processors.

Processing group 68G:

Using 24 processors.

Processing group 4c:
691 177 514
Total number of sequences before pre.cluster was 691.
pre.cluster removed 514 sequences.

It took 0 secs to cluster 691 sequences.
```

```
Processing group 50G:

Using 24 processors.

Processing group 52G:

Using 24 processors.

Processing group 55G:

Using 24 processors.

Processing group 14b:
8 8 0
Total number of sequences before pre.cluster was 8.
pre.cluster removed 0 sequences.

It took 0 secs to cluster 8 sequences.

Processing group 14c:
301 77 224
Total number of sequences before pre.cluster was 301.
pre.cluster removed 224 sequences.

It took 0 secs to cluster 301 sequences.

Processing group 15b:
2 2 0
Total number of sequences before pre.cluster was 2.
pre.cluster removed 0 sequences.

It took 0 secs to cluster 2 sequences.

Using 24 processors.

Processing group 3c:
1799 430 1369
Total number of sequences before pre.cluster was 1799.
pre.cluster removed 1369 sequences.

It took 0 secs to cluster 1799 sequences.

Processing group 40G:

Using 24 processors.

Processing group 2b:
14 10 4
Total number of sequences before pre.cluster was 14.
pre.cluster removed 4 sequences.

It took 0 secs to cluster 14 sequences.

Processing group 2c:
445 102 343
Total number of sequences before pre.cluster was 445.
pre.cluster removed 343 sequences.

It took 0 secs to cluster 445 sequences.

Processing group 31G:
316 70 246
Total number of sequences before pre.cluster was 316.
pre.cluster removed 246 sequences.

It took 0 secs to cluster 316 sequences.

Using 24 processors.
```

```
Processing group 8c:
643 133 510
Total number of sequences before pre.cluster was 643.
pre.cluster removed 510 sequences.

It took 0 secs to cluster 643 sequences.

Processing group 9b:
5 5 0
Total number of sequences before pre.cluster was 5.
pre.cluster removed 0 sequences.

It took 0 secs to cluster 5 sequences.

Processing group 9c:
24627 1792 22835
Total number of sequences before pre.cluster was 24627.
pre.cluster removed 22835 sequences.

It took 3 secs to cluster 24627 sequences.

Processing group 49G:
3537 778 2759
Total number of sequences before pre.cluster was 3537.
pre.cluster removed 2759 sequences.

It took 1 secs to cluster 3537 sequences.

Using 24 processors.

Processing group 42G:
23660 1749 21911
Total number of sequences before pre.cluster was 23660.
pre.cluster removed 21911 sequences.

It took 2 secs to cluster 23660 sequences.

Processing group 53G:
5923 599 5324
Total number of sequences before pre.cluster was 5923.
pre.cluster removed 5324 sequences.

It took 0 secs to cluster 5923 sequences.

Processing group 4b:
20 10 10
Total number of sequences before pre.cluster was 20.
pre.cluster removed 10 sequences.

It took 0 secs to cluster 20 sequences.
869 122 747
Total number of sequences before pre.cluster was 869.
pre.cluster removed 747 sequences.

It took 0 secs to cluster 869 sequences.

Processing group 54G:
22402 2012 20390
Total number of sequences before pre.cluster was 22402.
pre.cluster removed 20390 sequences.

It took 3 secs to cluster 22402 sequences.

Processing group 56G:

Using 24 processors.
```

```
Processing group 62G:

Using 24 processors.

Processing group 65G:
282 71 211
Total number of sequences before pre.cluster was 282.
pre.cluster removed 211 sequences.

It took 0 secs to cluster 282 sequences.

Processing group 66G:

Using 24 processors.

Processing group 32G:
4736 282 4454
Total number of sequences before pre.cluster was 4736.
pre.cluster removed 4454 sequences.

It took 0 secs to cluster 4736 sequences.

Processing group 33G:

Using 24 processors.

Processing group 35G:
93 21 72
Total number of sequences before pre.cluster was 93.
pre.cluster removed 72 sequences.

It took 0 secs to cluster 93 sequences.

Processing group 36G:
1296 255 1041
Total number of sequences before pre.cluster was 1296.
pre.cluster removed 1041 sequences.

It took 1 secs to cluster 1296 sequences.

Processing group 34G:
220 39 181
Total number of sequences before pre.cluster was 220.
pre.cluster removed 181 sequences.

It took 0 secs to cluster 220 sequences.
21977 2151 19826
Total number of sequences before pre.cluster was 21977.
pre.cluster removed 19826 sequences.

21991 2000 19991
Total number of sequences before pre.cluster was 21991.
pre.cluster removed 19991 sequences.

It took 5 secs to cluster 21977 sequences.

Processing group 51G:
It took 4 secs to cluster 21991 sequences.

Processing group 41G:
419 52 367
Total number of sequences before pre.cluster was 419.
pre.cluster removed 367 sequences.

It took 0 secs to cluster 419 sequences.
2897 243 2654
Total number of sequences before pre.cluster was 2897.
pre.cluster removed 2654 sequences.
```

```
It took 0 secs to cluster 2897 sequences.

Using 24 processors.

Processing group 38G:

Using 24 processors.

Processing group 45G:
7895 578 7317
Total number of sequences before pre.cluster was 7895.
pre.cluster removed 7317 sequences.

It took 0 secs to cluster 7895 sequences.

Processing group 39G:
657 94 563
Total number of sequences before pre.cluster was 657.
pre.cluster removed 563 sequences.

It took 0 secs to cluster 657 sequences.

Processing group 3b:
61 32 29
Total number of sequences before pre.cluster was 61.
pre.cluster removed 29 sequences.

It took 0 secs to cluster 61 sequences.
10636 974 9662
Total number of sequences before pre.cluster was 10636.
pre.cluster removed 9662 sequences.

It took 1 secs to cluster 10636 sequences.

Processing group 37G:
118 28 90
Total number of sequences before pre.cluster was 118.
pre.cluster removed 90 sequences.

It took 0 secs to cluster 118 sequences.
9964 1107 8857
Total number of sequences before pre.cluster was 9964.
pre.cluster removed 8857 sequences.

It took 2 secs to cluster 9964 sequences.

Processing group 67G:
928 141 787
Total number of sequences before pre.cluster was 928.
pre.cluster removed 787 sequences.

It took 0 secs to cluster 928 sequences.

Using 24 processors.

Processing group 15c:
555 113 442
Total number of sequences before pre.cluster was 555.
pre.cluster removed 442 sequences.

It took 0 secs to cluster 555 sequences.

Processing group 1b:
14 12 2
Total number of sequences before pre.cluster was 14.
pre.cluster removed 2 sequences.
```

```
It took 0 secs to cluster 14 sequences.

Processing group 1c:
14201 1514 12687
Total number of sequences before pre.cluster was 14201.
pre.cluster removed 12687 sequences.


Using 24 processors.

Processing group 13b:
4 3 1
Total number of sequences before pre.cluster was 4.
pre.cluster removed 1 sequences.

It took 0 secs to cluster 4 sequences.

Processing group 13c:
It took 3 secs to cluster 14201 sequences.

Processing group 43G:
548 75 473
Total number of sequences before pre.cluster was 548.
pre.cluster removed 473 sequences.

It took 0 secs to cluster 548 sequences.

Processing group 44G:
2016 378 1638
Total number of sequences before pre.cluster was 2016.
pre.cluster removed 1638 sequences.

It took 0 secs to cluster 2016 sequences.
2156 451 1705
Total number of sequences before pre.cluster was 2156.
pre.cluster removed 1705 sequences.

It took 0 secs to cluster 2156 sequences.
8794 991 7803
Total number of sequences before pre.cluster was 8794.
pre.cluster removed 7803 sequences.

It took 1 secs to cluster 8794 sequences.

Processing group 46G:

Using 24 processors.

Processing group 58G:
4025 472 3553
Total number of sequences before pre.cluster was 4025.
pre.cluster removed 3553 sequences.

It took 0 secs to cluster 4025 sequences.

Processing group 59G:
1316 165 1151
Total number of sequences before pre.cluster was 1316.
pre.cluster removed 1151 sequences.

It took 1 secs to cluster 1316 sequences.

Processing group 5b:
3 3 0
Total number of sequences before pre.cluster was 3.
pre.cluster removed 0 sequences.

It took 0 secs to cluster 3 sequences.
```

```
Using 24 processors.

Processing group 12b:
25 12 13
Total number of sequences before pre.cluster was 25.
pre.cluster removed 13 sequences.

It took 0 secs to cluster 25 sequences.

Processing group 12c:
2128 617 1511
Total number of sequences before pre.cluster was 2128.
pre.cluster removed 1511 sequences.

It took 0 secs to cluster 2128 sequences.

Using 24 processors.

Processing group 10b:
10 7 3
Total number of sequences before pre.cluster was 10.
pre.cluster removed 3 sequences.

It took 0 secs to cluster 10 sequences.

Processing group 10c:
548 188 360
Total number of sequences before pre.cluster was 548.
pre.cluster removed 360 sequences.

It took 0 secs to cluster 548 sequences.
25441 2349 23092
Total number of sequences before pre.cluster was 25441.
pre.cluster removed 23092 sequences.


Using 24 processors.

Processing group 11b:
33 16 17
Total number of sequences before pre.cluster was 33.
pre.cluster removed 17 sequences.

It took 0 secs to cluster 33 sequences.

Processing group 11c:
It took 3 secs to cluster 25441 sequences.

Processing group 57G:
3059 966 2093
Total number of sequences before pre.cluster was 3059.
pre.cluster removed 2093 sequences.

It took 1 secs to cluster 3059 sequences.
6016 987 5029
Total number of sequences before pre.cluster was 6016.
pre.cluster removed 5029 sequences.

It took 1 secs to cluster 6016 sequences.
20279 1723 18556
Total number of sequences before pre.cluster was 20279.
pre.cluster removed 18556 sequences.

It took 2 secs to cluster 20279 sequences.

Processing group 47G:
1608 261 1347
```

```
Total number of sequences before pre.cluster was 1608.
pre.cluster removed 1347 sequences.

It took 0 secs to cluster 1608 sequences.
23136 2335 20801
Total number of sequences before pre.cluster was 23136.
pre.cluster removed 20801 sequences.

It took 3 secs to cluster 23136 sequences.
30912 3591 27321
Total number of sequences before pre.cluster was 30912.
pre.cluster removed 27321 sequences.

It took 11 secs to cluster 30912 sequences.

Processing group 6b:
1 1 0
Total number of sequences before pre.cluster was 1.
pre.cluster removed 0 sequences.

It took 0 secs to cluster 1 sequences.

Processing group 6c:
204 63 141
Total number of sequences before pre.cluster was 204.
pre.cluster removed 141 sequences.

It took 0 secs to cluster 204 sequences.
36979 3532 33447
Total number of sequences before pre.cluster was 36979.
pre.cluster removed 33447 sequences.

It took 9 secs to cluster 36979 sequences.
36259 3885 32374
Total number of sequences before pre.cluster was 36259.
pre.cluster removed 32374 sequences.

It took 12 secs to cluster 36259 sequences.

Processing group 61G:
2366 263 2103
Total number of sequences before pre.cluster was 2366.
pre.cluster removed 2103 sequences.

It took 0 secs to cluster 2366 sequences.
44502 4727 39775
Total number of sequences before pre.cluster was 44502.
pre.cluster removed 39775 sequences.

It took 13 secs to cluster 44502 sequences.

Processing group 63G:
469 116 353
Total number of sequences before pre.cluster was 469.
pre.cluster removed 353 sequences.

It took 0 secs to cluster 469 sequences.

Processing group 64G:
29289 3014 26275
Total number of sequences before pre.cluster was 29289.
pre.cluster removed 26275 sequences.

It took 5 secs to cluster 29289 sequences.
It took 62 secs to run pre.cluster.

Output File Names:
stability.trim.contigs.good.unique.good.filter.unique.precluster.fasta
```

```
stability.trim.contigs.good.unique.good.filter.unique.precluster.count_table
stability.trim.contigs.good.unique.good.filter.unique.precluster.10b.map
stability.trim.contigs.good.unique.good.filter.unique.precluster.10c.map
stability.trim.contigs.good.unique.good.filter.unique.precluster.11b.map
stability.trim.contigs.good.unique.good.filter.unique.precluster.11c.map
stability.trim.contigs.good.unique.good.filter.unique.precluster.12b.map
stability.trim.contigs.good.unique.good.filter.unique.precluster.12c.map
stability.trim.contigs.good.unique.good.filter.unique.precluster.13b.map
stability.trim.contigs.good.unique.good.filter.unique.precluster.13c.map
stability.trim.contigs.good.unique.good.filter.unique.precluster.14b.map
stability.trim.contigs.good.unique.good.filter.unique.precluster.14c.map
stability.trim.contigs.good.unique.good.filter.unique.precluster.15b.map
stability.trim.contigs.good.unique.good.filter.unique.precluster.15c.map
stability.trim.contigs.good.unique.good.filter.unique.precluster.1b.map
stability.trim.contigs.good.unique.good.filter.unique.precluster.1c.map
stability.trim.contigs.good.unique.good.filter.unique.precluster.2b.map
stability.trim.contigs.good.unique.good.filter.unique.precluster.2c.map
stability.trim.contigs.good.unique.good.filter.unique.precluster.31G.map
stability.trim.contigs.good.unique.good.filter.unique.precluster.32G.map
stability.trim.contigs.good.unique.good.filter.unique.precluster.33G.map
stability.trim.contigs.good.unique.good.filter.unique.precluster.34G.map
stability.trim.contigs.good.unique.good.filter.unique.precluster.35G.map
stability.trim.contigs.good.unique.good.filter.unique.precluster.36G.map
stability.trim.contigs.good.unique.good.filter.unique.precluster.37G.map
stability.trim.contigs.good.unique.good.filter.unique.precluster.38G.map
stability.trim.contigs.good.unique.good.filter.unique.precluster.39G.map
stability.trim.contigs.good.unique.good.filter.unique.precluster.3b.map
stability.trim.contigs.good.unique.good.filter.unique.precluster.3c.map
stability.trim.contigs.good.unique.good.filter.unique.precluster.40G.map
stability.trim.contigs.good.unique.good.filter.unique.precluster.41G.map
stability.trim.contigs.good.unique.good.filter.unique.precluster.42G.map
stability.trim.contigs.good.unique.good.filter.unique.precluster.43G.map
stability.trim.contigs.good.unique.good.filter.unique.precluster.44G.map
stability.trim.contigs.good.unique.good.filter.unique.precluster.45G.map
stability.trim.contigs.good.unique.good.filter.unique.precluster.46G.map
stability.trim.contigs.good.unique.good.filter.unique.precluster.47G.map
stability.trim.contigs.good.unique.good.filter.unique.precluster.48G.map
stability.trim.contigs.good.unique.good.filter.unique.precluster.49G.map
stability.trim.contigs.good.unique.good.filter.unique.precluster.4b.map
stability.trim.contigs.good.unique.good.filter.unique.precluster.4c.map
stability.trim.contigs.good.unique.good.filter.unique.precluster.50G.map
stability.trim.contigs.good.unique.good.filter.unique.precluster.51G.map
stability.trim.contigs.good.unique.good.filter.unique.precluster.52G.map
stability.trim.contigs.good.unique.good.filter.unique.precluster.53G.map
stability.trim.contigs.good.unique.good.filter.unique.precluster.54G.map
stability.trim.contigs.good.unique.good.filter.unique.precluster.55G.map
stability.trim.contigs.good.unique.good.filter.unique.precluster.56G.map
stability.trim.contigs.good.unique.good.filter.unique.precluster.57G.map
stability.trim.contigs.good.unique.good.filter.unique.precluster.58G.map
stability.trim.contigs.good.unique.good.filter.unique.precluster.59G.map
stability.trim.contigs.good.unique.good.filter.unique.precluster.5b.map
stability.trim.contigs.good.unique.good.filter.unique.precluster.5c.map
stability.trim.contigs.good.unique.good.filter.unique.precluster.60G.map
stability.trim.contigs.good.unique.good.filter.unique.precluster.61G.map
stability.trim.contigs.good.unique.good.filter.unique.precluster.62G.map
stability.trim.contigs.good.unique.good.filter.unique.precluster.63G.map
stability.trim.contigs.good.unique.good.filter.unique.precluster.64G.map
stability.trim.contigs.good.unique.good.filter.unique.precluster.65G.map
stability.trim.contigs.good.unique.good.filter.unique.precluster.66G.map
stability.trim.contigs.good.unique.good.filter.unique.precluster.67G.map
stability.trim.contigs.good.unique.good.filter.unique.precluster.68G.map
stability.trim.contigs.good.unique.good.filter.unique.precluster.6b.map
stability.trim.contigs.good.unique.good.filter.unique.precluster.6c.map
stability.trim.contigs.good.unique.good.filter.unique.precluster.7b.map
stability.trim.contigs.good.unique.good.filter.unique.precluster.7c.map
stability.trim.contigs.good.unique.good.filter.unique.precluster.8b.map
stability.trim.contigs.good.unique.good.filter.unique.precluster.8c.map
stability.trim.contigs.good.unique.good.filter.unique.precluster.9b.map
```

```
stability.trim.contigs.good.unique.good.filter.unique.precluster.9c.map


mothur > summary.seqs(fasta=current, count=current)
Using stability.trim.contigs.good.unique.good.filter.unique.precluster.count_table as input file for
the count parameter.
Using stability.trim.contigs.good.unique.good.filter.unique.precluster.fasta as input file for the
fasta parameter.

Using 24 processors.

Start End NBases Ambigs Polymer NumSeqs
Minimum: 1 890 434 0 3 1
2.5%-tile: 1 890 441 0 3 26653
25%-tile: 1 890 464 0 4 266521
Median: 1 890 464 0 5 533042
75%-tile: 1 890 464 0 5 799562
97.5%-tile: 1 890 464 0 6 1039430
Maximum: 1 890 469 0 9 1066082
Mean: 1 890 460.79 0 4.59241
# of unique seqs: 49196
total # of seqs: 1066082

Output File Names:
stability.trim.contigs.good.unique.good.filter.unique.precluster.summary

It took 0 secs to summarize 1066082 sequences.

mothur > split.abund(fasta=current, count=current, cutoff=5)
Using stability.trim.contigs.good.unique.good.filter.unique.precluster.count_table as input file for
the count parameter.
Using stability.trim.contigs.good.unique.good.filter.unique.precluster.fasta as input file for the
fasta parameter.

Output File Names:
stability.trim.contigs.good.unique.good.filter.unique.precluster.rare.count_table
stability.trim.contigs.good.unique.good.filter.unique.precluster.abund.count_table
stability.trim.contigs.good.unique.good.filter.unique.precluster.rare.fasta
stability.trim.contigs.good.unique.good.filter.unique.precluster.abund.fasta


mothur >
summary.seqs(fasta=stability.trim.contigs.good.unique.good.filter.unique.precluster.abund.fasta,
count=stability.trim.contigs.good.unique.good.filter.unique.precluster.abund.count_table,
processors=24)

Using 24 processors.

Start End NBases Ambigs Polymer NumSeqs
Minimum: 1 890 437 0 3 1
2.5%-tile: 1 890 442 0 3 25217
25%-tile: 1 890 464 0 4 252166
Median: 1 890 464 0 5 504331
75%-tile: 1 890 464 0 5 756496
97.5%-tile: 1 890 464 0 6 983445
Maximum: 1 890 465 0 8 1008661
Mean: 1 890 460.78 0 4.58481
# of unique seqs: 1951
total # of seqs: 1008661

Output File Names:
stability.trim.contigs.good.unique.good.filter.unique.precluster.abund.summary

It took 0 secs to summarize 1008661 sequences.

mothur >
classify.seqs(fasta=stability.trim.contigs.good.unique.good.filter.unique.precluster.abund.fasta,
count=stability.trim.contigs.good.unique.good.filter.unique.precluster.abund.count_table,
```

```
reference=gg_13_5_99.fasta, taxonomy=gg_13_5_99.gg.tax, cutoff=80)

Using 24 processors.
Generating search database... DONE.
It took 104 seconds generate search database.

Reading in the gg_13_5_99.gg.tax taxonomy... DONE.
Calculating template taxonomy tree... DONE.
Calculating template probabilities... DONE.
It took 317 seconds get probabilities.
Classifying sequences from
stability.trim.contigs.good.unique.good.filter.unique.precluster.abund.fasta ...

It took 9 secs to classify 1951 sequences.


It took 1 secs to create the summary file for 1951 sequences.


Output File Names:
stability.trim.contigs.good.unique.good.filter.unique.precluster.abund.gg.wang.taxonomy
stability.trim.contigs.good.unique.good.filter.unique.precluster.abund.gg.wang.tax.summary


mothur > summary.seqs(fasta=current, count=current)
Using stability.trim.contigs.good.unique.good.filter.unique.precluster.abund.count_table as input
file for the count parameter.
Using stability.trim.contigs.good.unique.good.filter.unique.precluster.abund.fasta as input file for
the fasta parameter.

Using 24 processors.

Start End NBases Ambigs Polymer NumSeqs
Minimum: 1 890 437 0 3 1
2.5%-tile: 1 890 442 0 3 25217
25%-tile: 1 890 464 0 4 252166
Median: 1 890 464 0 5 504331
75%-tile: 1 890 464 0 5 756496
97.5%-tile: 1 890 464 0 6 983445
Maximum: 1 890 465 0 8 1008661
Mean: 1 890 460.78 0 4.58481
# of unique seqs: 1951
total # of seqs: 1008661

Output File Names:
stability.trim.contigs.good.unique.good.filter.unique.precluster.abund.summary

It took 0 secs to summarize 1008661 sequences.

mothur > dist.seqs(fasta=current, cutoff=0.03, processors=24)
Using stability.trim.contigs.good.unique.good.filter.unique.precluster.abund.fasta as input file for
the fasta parameter.

Using 24 processors.

Output File Names:
stability.trim.contigs.good.unique.good.filter.unique.precluster.abund.dist

It took 1 seconds to calculate the distances for 1951 sequences.

mothur > cluster(column=current, count=current, cutoff=0.03)
Using stability.trim.contigs.good.unique.good.filter.unique.precluster.abund.dist as input file for
the column parameter.
Using stability.trim.contigs.good.unique.good.filter.unique.precluster.abund.count_table as input
file for the count parameter.

Using 24 processors.
```

```
Clustering stability.trim.contigs.good.unique.good.filter.unique.precluster.abund.dist


iter time label num_otus cutoff tp tn fp fn sensitivity specificity ppv npv fdr accuracy mcc f1score
0 0 0.03 1951 0.03 0 1819916 0 82309 0 1 0 0.95673 0 0.95673 0 0
1 0 0.03 460 0.03 80472 1819223 693 1837 0.977682 0.999619 0.991462 0.998991 0.00853816 0.99867
0.983855 0.984524
2 0 0.03 447 0.03 80561 1819215 701 1748 0.978763 0.999615 0.991374 0.99904 0.00862642 0.998713
0.984377 0.985028
3 0 0.03 447 0.03 80561 1819215 701 1748 0.978763 0.999615 0.991374 0.99904 0.00862642 0.998713
0.984377 0.985028


It took 1 seconds to cluster

Output File Names:
stability.trim.contigs.good.unique.good.filter.unique.precluster.abund.opti_mcc.list
stability.trim.contigs.good.unique.good.filter.unique.precluster.abund.opti_mcc.steps
stability.trim.contigs.good.unique.good.filter.unique.precluster.abund.opti_mcc.sensspec


mothur > make.shared(list=current, count=current, label=0.03)
Using stability.trim.contigs.good.unique.good.filter.unique.precluster.abund.count_table as input
file for the count parameter.
Using stability.trim.contigs.good.unique.good.filter.unique.precluster.abund.opti_mcc.list as input
file for the list parameter.
0.03

Output File Names:
stability.trim.contigs.good.unique.good.filter.unique.precluster.abund.opti_mcc.shared


mothur > classify.otu(list=current, count=current, taxonomy=current, label=0.03)
Using stability.trim.contigs.good.unique.good.filter.unique.precluster.abund.count_table as input
file for the count parameter.
Using stability.trim.contigs.good.unique.good.filter.unique.precluster.abund.opti_mcc.list as input
file for the list parameter.
Using stability.trim.contigs.good.unique.good.filter.unique.precluster.abund.gg.wang.taxonomy as
input file for the taxonomy parameter.
0.03 447

Output File Names:
stability.trim.contigs.good.unique.good.filter.unique.precluster.abund.opti_mcc.0.03.cons.taxonomy
stability.trim.contigs.good.unique.good.filter.unique.precluster.abund.opti_mcc.0.03.cons.tax.summary


mothur > system(mv
stability.trim.contigs.good.unique.good.filter.unique.precluster.abund.opti_mcc.shared
stability.an.shared)


mothur > system(mv
stability.trim.contigs.good.unique.good.filter.unique.precluster.abund.opti_mcc.0.03.cons.taxonomy
stability.an.cons.taxonomy)


mothur > count.groups(shared=stability.an.shared)
10c contains 380.
11b contains 15.
11c contains 2286.
12b contains 8.
12c contains 1619.
13c contains 1950.
14c contains 239.
15c contains 492.
1b contains 3.
1c contains 2012.
2c contains 377.
```

```
31G contains 286.
32G contains 11407.
33G contains 1307.
34G contains 246.
35G contains 83.
36G contains 23130.
37G contains 105.
38G contains 19023.
39G contains 707.
3b contains 24.
3c contains 1515.
40G contains 50376.
41G contains 473.
42G contains 29800.
43G contains 639.
44G contains 60795.
45G contains 10885.
46G contains 51090.
47G contains 1629.
48G contains 60917.
49G contains 7509.
4b contains 6.
4c contains 567.
50G contains 46085.
51G contains 3973.
52G contains 56637.
53G contains 939.
54G contains 94606.
55G contains 41453.
56G contains 53325.
57G contains 7206.
58G contains 5933.
59G contains 1432.
5c contains 1666.
60G contains 79453.
61G contains 2976.
62G contains 102875.
63G contains 411.
64G contains 77692.
65G contains 215.
66G contains 20335.
67G contains 1248.
68G contains 64142.
6c contains 150.
8c contains 585.
9c contains 3424.

Total seqs: 1008661.

Output File Names:
stability.an.count.summary


mothur > get.oturep(column=current, list=current, fasta=current, count=current, label=0.03)
Using stability.trim.contigs.good.unique.good.filter.unique.precluster.abund.dist as input file for the column parameter.
Using stability.trim.contigs.good.unique.good.filter.unique.precluster.abund.count_table as input file for the count parameter.
Using stability.trim.contigs.good.unique.good.filter.unique.precluster.abund.fasta as input file for the fasta parameter.
Using stability.trim.contigs.good.unique.good.filter.unique.precluster.abund.opti_mcc.list as input file for the list parameter.
********************#****#****#****#****#****#****#****#****#****#
Reading matrix: |||||||||||||||||||||||||||||||||||||||||||||||||||
***********************************************************************
0.03 447

Output File Names:
```

```
stability.trim.contigs.good.unique.good.filter.unique.precluster.abund.opti_mcc.0.03.rep.count_table
stability.trim.contigs.good.unique.good.filter.unique.precluster.abund.opti_mcc.0.03.rep.fasta


mothur > make.biom(shared=stability.an.shared, label=0.03, reftaxonomy=gg_13_5_99.gg.tax,
constaxonomy=stability.an.cons.taxonomy, picrust=97_otu_map.txt)
0.03

Output File Names:
stability.an.0.03.biom
stability.an.0.03.biom_shared


mothur > quit()


<<<<<<<<<<<<<<<<<<<<<<<<<<<<<<^>>>>>>>>>>>>>>>>>>>>>>>>>>>>>
<<<<<<<<<<<<<<<<<<<<<<<<<<<<<<^>>>>>>>>>>>>>>>>>>>>>>>>>>>>>
<<<<<<<<<<<<<<<<<<<<<<<<<<<<<<^>>>>>>>>>>>>>>>>>>>>>>>>>>>>>
Detected 2 [WARNING] messages, please review.
<<<<<<<<<<<<<<<<<<<<<<<<<<<<<<^>>>>>>>>>>>>>>>>>>>>>>>>>>>>>
<<<<<<<<<<<<<<<<<<<<<<<<<<<<<<^>>>>>>>>>>>>>>>>>>>>>>>>>>>>>
<<<<<<<<<<<<<<<<<<<<<<<<<<<<<<^>>>>>>>>>>>>>>>>>>>>>>>>>>>>>
```
